# Editorial: Antibiotic potentiators against drug-resistant pathogens: Discovery, development and clinical applications

**DOI:** 10.3389/fmicb.2023.1173906

**Published:** 2023-03-07

**Authors:** Bhabatosh Das, Dinesh Mahajan, Jasna Rakonjac

**Affiliations:** ^1^Centre for Bacterial Diseases and Antibiotic Resistance Research, Translational Health Science and Technology Institute, Faridabad, India; ^2^Chemistry and Pharmacology Lab, Centre for Drug Design and Discovery, Translational Health Science and Technology Institute, Faridabad, India; ^3^College of Sciences, Massey University, Palmerston North, New Zealand

**Keywords:** antibiotics, potentiators, resistance, inhibitors, plasmids

## Introduction

Antimicrobial resistance (AMR) is a natural evolutionary consequence induced by massive antibiotic usage. The significantly higher rate of AMR and its spread is largely attributed to the illicit use and management of antibiotics, their production, and residual disposal in the environment (Das et al., 2017). AMR-associated and -contributed deaths are expected to exceed 4.5 million in 2023 due to the global spread of AMR in clinically important pathogens (Antimicrobial Resistance Collaborators, 2022). In addition, the issue of AMR is also plaguing the management of other clinical conditions such as organ transplantation, microbiome transplantation, and inflammation management in chronic metabolic diseases leading to poor success rates. To tackle the AMR crisis and identify novel antibiotics or alternatives to antibiotics, several global initiatives are at different stages of completion (Narendrakumar et al., 2022). The current special issue covers updated research initiatives expanding the toolset against AMR pathogens by including (i) repurposed, already approved, drugs normally used for the treatment of non-infectious diseases; (ii) antibiotic potentiators that are not antibacterials, but rather block the resistance functions or increase membrane permeability, thus enhance antibiotics' activity; (iii) synergistic combinations of antibiotics; (iv) alternative therapies to minimize antibiotic uses.

## Restoring antibacterial activity by potentiators

Restoring the antibacterial activity of clinically important antibiotics that lost their efficacy over the years because of the reduced uptake by pathogens, or spread of specific resistance functions in organisms by horizontal gene transfer is of great public health importance. Chawla et al. summarized the current status around the discovery and development of potentiators and their modes of action against antibiotic-resistant pathogens. The non-antibiotic compounds that re-sensitize resistant pathogens are known as potentiators or antibiotic adjuvants. Potentiators only work in conjunction with antibiotics and enhance their efficacy against a resistant pathogen. Authors deliberated on the different possible modes of action for the potentiators such as direct inhibition of resistance enzymes or efflux pumps or reducing the stability, inheritance, and dissemination of the vectors that carry the resistance genes. Both natural and synthetic compounds have been harnessed for the discovery and development of novel antibiotic potentiators. The β-lactamase inhibitors are the most commonly used potentiators in clinical practice.

The work of Kumar et al. mainly focused on the discovery and development of a compound possessing anti-biofilm and potentiation activity. Authors reported that the guanidine derivative of silver could act as a colistin potentiator against multidrug-resistant *Acinetobacter baumannii* and other Gram-negative colistin-resistant pathogens. The nano-formulation, comprising silver nanoparticles and self-assembled guanidinium, also has bactericidal activity against planktonic and biofilm-associated microbes. The identified compound helps colistin interrupt the integrity of bacterial cell membranes. Such compounds can potentially be used to sterilize surgical equipment and the surfaces of the intensive care unit, where multidrug-resistant pathogens are of primary clinical concern.

Liu et al. discovered an efflux pump inhibitor, phenylalanine-arginine-naphthylamide (PAβN), which was found to increase the antimicrobial activity of the aminoglycoside antibiotic neomycin in a duck pathogen, *Riemerella anatipestifer*, a Gram-negative bacterium that causes septicemia. PAβN competes with neomycin for the same efflux pump, increasing neomycin's efficacy against aminoglycoside-resistant *R. anatipestifer*. The *in vitro* and *in vivo* experimental observations indicate PAβN can potentiate the activity of neomycin against neomycin-resistant *R. anatipestifer*.

## Identification of antibiotics combination exerting a synergistic effect

A synergistic effect between structurally different antibiotics can substantially improve the effectiveness of antimicrobial agents that, if used alone, merely retards the growth of a resistant pathogen. Such combinational therapy may potentially restrict the emergence and spread of resistant pathogens. A study conducted by Al-Marzooq et al. investigated the synergistic effect of polymyxin B non-apeptide (PMBN), a polymyxin derivative, with the aminoglycoside group of the antibiotic azithromycin (AZT). In different strains of the Gram-negative model bacterium *Escherichia coli*, PMBN substantially improves the efficacy of AZT, although the standalone minimum inhibitory concentration (MIC) of AZT is very high in the analyzed strains. Their genetic analyses reveal that the PBN and AZT combinations can neutralize the resistance functions of *mphA*-encoded macrolide phosphotransferase; however, this synergistic effect is not significant in a strain carrying an additional *ermB* gene, which encodes macrolide methylase. The authors proposed that the PMBN increases AZT permeability by partially interfering with the outer membrane permeability of the tested E. coli strains.

The study conducted by Xu et al. focused on the synergistic activity of colistin and naringenin, a flavonoid belonging to the flavanones' subclass, against colistin-resistant Gram-negative pathogens that infect *Galleria mellonella*. Authors reported that the naringenin-colistin combination also inhibits biofilm formation and reduces the microbial load in the biofilm matrix. The synergistic antibacterial activity leads to increase membrane permeability due to the loss of outer membrane integrity, which facilitates the action of antibiotics to clear microbial infections in *G. mellonella*.

Mirzaei et al. investigated the synergistic effects of melittin, a 26 amino-acid basic peptide produced by the honeybee in their venom, with vancomycin and rifampin against multidrug-resistant methicillin-resistant *Staphylococcus epidermidis* (MDR-MRSE). Authors demonstrated that vancomycin- and rifampin-resistant MRSE showed sensitivity to both drugs when all three components were used together in combination. Mechanistic insights into the synergism of melittin, vancomycin, and rifampin are yet to be established. Importantly, Zhang et al. reported that drug resistance characteristics vary depending on the pathogens, and geography which leads to an additional level of complexity. Thus the development of synergistic antibiotics for any resistant pathogen needs regional or global multi-center validation.

## Alternative to antibiotics

Although antibiotics are the first choice of clinicians for treating bacterial infections, with the emergence of extensive drug-resistant pathogens, researchers have found several alternatives to kill microbial pathogens (bacteriophages), attenuate their virulence (antivirulents), and prevent infections (vaccines). Yuan et al. reported repurposing dimetridazole and ribavirin approved for the treatment of protozoans and viral infections, respectively, to attenuate Pseudomonas aeruginosa virulence. Using a Caenorhabditis elegans model system, the authors demonstrated that dimetridazole and ribavirin reduce P. aeruginosa virulence, particularly protease production, and biofilm formation by modulating its quorum sensing system.

## Perspective

**Figure 1:**
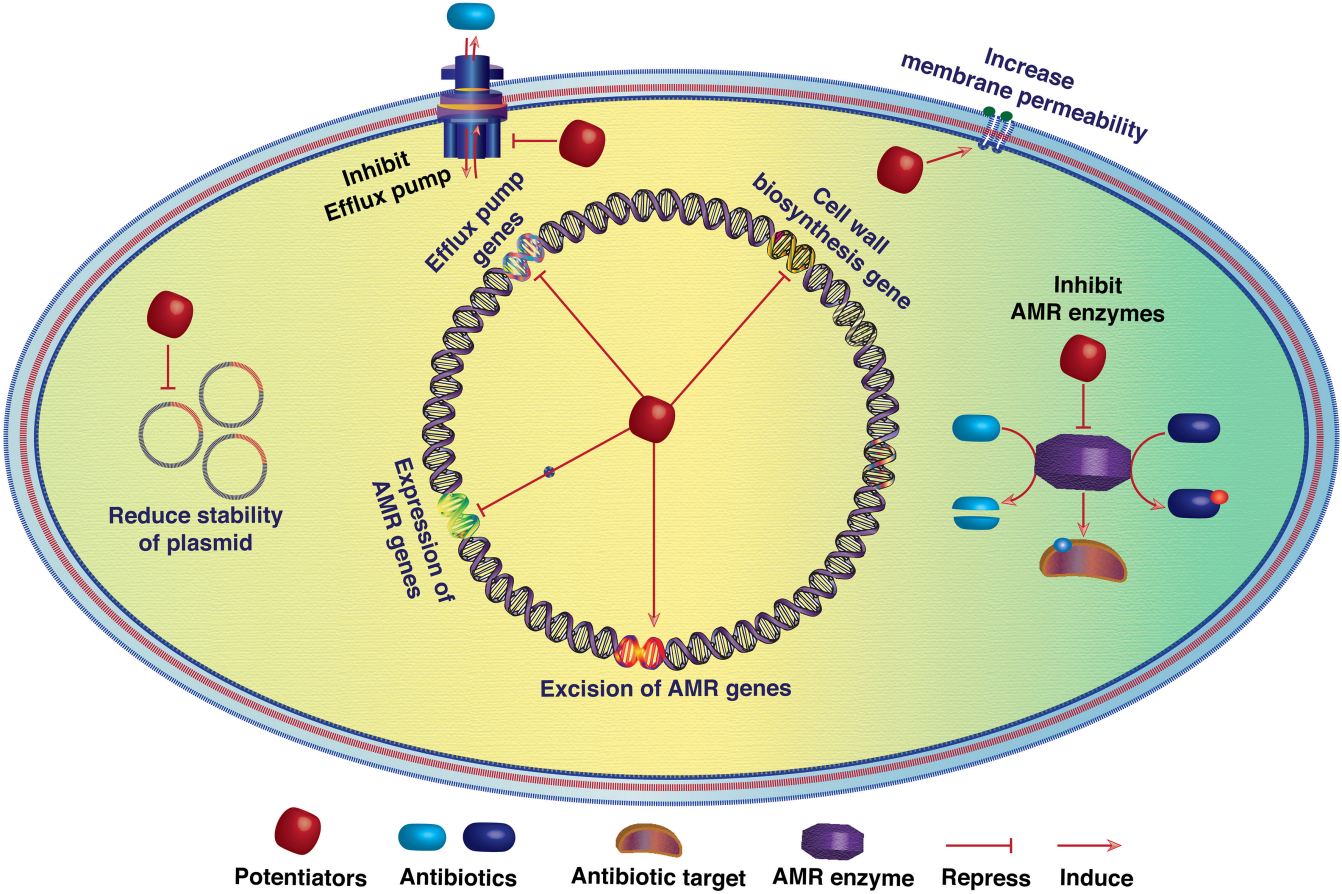
Schematic diagram showing the potential targets of antibiotic potentiators. Potentiators may directly inhibit the activity of antibiotic resistance enzymes or reduce the activity of efflux pumps. Furthermore, by modulating the expression of antibiotic resistance genes (ARGs), potentiators may decrease their cellular level and resistance functions. Increasing membrane permeability may also aid antibiotics in killing resistant bacteria by reaching the threshold level. Several mobile genetic elements (MGEs) such as plasmids, integrative conjugative elements, and transposons are linked with ARGs. Thus, antibiotic resistance reversion is also possible by reducing the stability of such MGEs.

Repeated exposure to sublethal doses of antibiotics leads to genetic changes and is the primary driver for the emergence of resistance among microbes. However, genetic traits that confirm AMR are extremely heterogeneous in different bacteria (Das et al., 2020). No resistance trait is widely conserved (Darby et al., 2022). While drug-resistant intracellular pathogens emerge primarily as a result of spontaneous mutations in the target gene (Mehrotra et al., 2021; Kumar et al., 2022), AMR in extracellular pathogens is primarily driven by the horizontal acquisition of resistance genes (Kumar et al., 2017). Thus, the development of antibiotic potentiators to neutralize resistance functions necessitates extensive genomic surveillance for appropriate target identification and pathogen-specific approaches for the development of new potentiators or anti-virulence drugs. Currently, only a few potentiators that inhibit beta-lactamase functions are being used in clinics. However, potentiators can be developed against several target molecules, including (i) resistance enzymes, (ii) efflux pumps, (iii) transcriptional factors that modulate the expression of resistance-associated genes, and (iv) functions that maintain the stability of resistance-linked mobile genetic elements (Figure 1). The articles and reviews of this special topic will provide thought-provoking introductions to drug discovery against resistant pathogens and will be an invaluable source of information for clinicians and basic scientists working to combat the resistance crisis.

## Author contributions

BD has written the original draft and finalized the editorial. DM and JR edited the write-up. All authors contributed to the article and approved the submitted version.
